# Trigger related outcomes of takotsubo syndrome in a cancer population

**DOI:** 10.3389/fcvm.2022.1019284

**Published:** 2022-10-28

**Authors:** Ayesha Safdar, Talha Ahmed, Victor Y. Liu, Antoine Addoumieh, Ali M. Agha, Dana E. Giza, Dinu V. Balanescu, Teodora Donisan, Tariq Dayah, Juan C. Lopez-Mattei, Peter Y. Kim, Saamir Hassan, Kaveh Karimzad, Nicolas Palaskas, January Y. Tsai, Gloria D. Iliescu, Eric H. Yang, Joerg Herrmann, Konstantinos Marmagkiolis, Paolo Angelini, Cezar A. Iliescu

**Affiliations:** ^1^Department of Medicine, Army Medical College, Rawalpindi, Pakistan; ^2^Department of Cardiology, The University of Texas MD Anderson Cancer Center, Houston, TX, United States; ^3^Department of Cardiovascular Medicine, The University of Texas Health Science Center at Houston, Houston, TX, United States; ^4^Department of Family and Community Medicine, McGovern Medical School at The University of Texas Health Science Center at Houston, Houston, TX, United States; ^5^Department of Anesthesiology and Perioperative Medicine, The University of Texas MD Anderson Cancer Center, Houston, TX, United States; ^6^Department of General Internal Medicine, The University of Texas MD Anderson Cancer Center, Houston, TX, United States; ^7^Department of Medicine, University of California, Los Angeles, Los Angeles, CA, United States; ^8^Division of Cardiovascular Medicine, Mayo Clinic, Rochester, MN, United States; ^9^Department of Cardiovascular Medicine, Florida Hospital Pepin Heart Institute, Tampa, FL, United States; ^10^Department of Cardiology, Texas Heart Institute, Houston, TX, United States

**Keywords:** takotsubo stress cardiomyopathy, chemotherapy, immunomodulators, cardio-oncology, takotsubo syndrome, triggers

## Abstract

**Background:**

Takotsubo syndrome (TTS) occurs more frequently in cancer patients than in the general population, but the effect of specific TTS triggers on outcomes in cancer patients is not well studied.

**Objectives:**

The study sought to determine whether triggering event (chemotherapy, immune-modulators vs. procedural or emotional stress) modifies outcomes in a cancer patient population with TTS.

**Methods:**

All cancer patients presenting with acute coronary syndrome (ACS) between December 2008 and December 2020 at our institution were enrolled in the catheterization laboratory registry. Demographic and clinical data of the identified patients with TTS were retrospective collected and further classified according to the TTS trigger. The groups were compared with regards to major adverse cardiac events, overall survival and recovery of left ventricular ejection fraction (LVEF) and global longitudinal strain (GLS) after TTS presentation.

**Results:**

Eighty one of the 373 cancer patients who presented with ACS met the Mayo criteria for TTS. The triggering event was determined to be “cancer specific triggers” (use of chemotherapy in 23, immunomodulators use in 7, and radiation in 4), and “traditional triggers” (medical triggers 22, and procedural 18 and emotional stress in 7). Of the 81 patients, 47 died, all from cancer-related causes (no cardiovascular mortality). Median survival was 11.9 months. Immunomodulator (IM) related TTS and radiation related TTS were associated with higher mortality during the follow-up. Patients with medical triggers showed the least recovery in LVEF and GLS while patients with emotional and chemotherapy triggers, showed the most improvement in LVEF and GLS, respectively.

**Conclusion:**

Cancer patients presenting with ACS picture have a high prevalence of TTS due to presence of traditional and cancer specific triggers. Survival and improvement in left ventricular systolic function seem to be related to the initial trigger for TTS.

## Introduction

Takotsubo syndrome (TTS) is a condition characterized by transient and reversible left ventricular (LV) systolic dysfunction frequently preceded by stressors, either emotional or physical; however, 28% of patients have no correlating evident trigger (1–4). Identifying the preceding event is important, as different triggers have been shown to influence TTS outcome (5, 6). A new classification of TTS was proposed recently to acknowledge these triggers: (1) primary TTS (TTS triggered by psychological stress) and (2) secondary TTS (related to physical factors such as surgery, trauma, and medical complications). Patients with secondary TTS have significantly worse prognosis than patients with primary TTS (6).

The trigger and underlying mechanisms of TTS are of particular importance in vulnerable populations, such as cancer patients in whom it is not only more prevalent but portrays a prognosis similar to true non-ST-elevation myocardial infarction (NSTEMI) (7–9). Among those with a history of TTS, cancer is the major cause of death, making cancer survivors with TTS an especially vulnerable population (9).

Patients with TTS often have increased tendencies to have thromboembolic events (10, 11) and often portray a worse prognosis in the setting of atrial fibrillation, malignant ventricular arrhythmias, and cardiogenic shock (12–14). A substantial number of TTS patients show an association with malignancy and some studies suggest that an appropriate screening for malignancy should be considered in these patients (15). Patients with TTS have more often malignant diseases than patients with MI and cancer patients with TTS have a worse clinical outcome (16). The underlying mechanism is unclear yet, but the results point at TTS being the syndrome of an extracardiac disease rather than a disease of cardiac origin (8). Positive emotional trigger related TTS has been described as “happy heart syndrome” (17). It is a rare type of TTS characterized by a higher prevalence of male patients and atypical, non-apical ballooning and similar outcomes compared to patients with negative stressors in short term studies (17).

The possible triggers of TTS in cancer patients can be both “cancer specific triggers” (use of chemotherapy, immunomodulators, and radiation) and “traditional triggers” (medical, procedural and emotional triggers). Several chemotherapeutic agents have been associated with TTS with varying hypothesized mechanisms but they have not been fully explored, and while a temporal relationship has been established, a causal relationship between chemotherapeutic/immune-modulating agents and TTS is yet to be confirmed (18). Due to aforementioned reasons, we hypothesize that a trigger-driven study may help improve understanding, guide treatment, and predict outcomes in a cancer patient population presenting with TTS.

## Materials and methods

### Study population

All cancer patients presenting with ACS who met the modified Mayo criteria for TTS (19, 20) and underwent quantitative coronary angiography and left ventriculogram between December 2008 and December 2020 at The University of Texas MD Anderson Cancer Center catheterization laboratory were entered into the laboratory registry. Demographic and clinical data was collected retrospectively. This study was approved by the Institutional Review Board. Patient informed consent was waived due to the retrospective nature of data analysis.

Baseline demographic information (age and sex), cardiovascular risk factors (coronary artery disease, hypertension, hyperlipidemia, diabetes mellitus, and smoking status), type of malignancy, cancer stage, and past and current anticancer therapeutic regimens were reviewed. Patients with multiple cancers were categorized according to the malignancy being actively treated at the time of TTS.

Potential sources of “traditional triggers” including emotional, procedural, or medical stress during the week prior to TTS onset were systematically reviewed. This review included terms referring to medical complications, any type of invasive procedure or surgery, and emotional challenges (anger, anxiety, or grief) as documented in the clinical notes before/during the TTS event.

Based on this information, patients were classified according to the predominant trigger as having chemotherapy-induced TTS (CI-TTS; chemotherapy), immunomodulator-induced TTS (IM-TTS: Immune checkpoint inhibitors, Notch Inhibitors, Colony stimulating factors, Anti CD-20) and procedural/emotional TTS (PE-TTS).

TTS was regarded as being chemotherapy/immunotherapy/radiation-induced if the episode occurred within one week after exposure to the trigger and no significant medical complication, invasive procedure, or significant emotional stress was noted during this time. This cut-off point is based on a recent review specifically on CI-TTS (21). Moreover, CI-TTS and IM-TTS were considered if cardiac symptoms were documented when chemotherapy and immunotherapy were administered respectively.

Imaging data including electrocardiograms (ECG), transthoracic echocardiograms, and coronary angiograms/left ventriculography as well as laboratory data including cardiac troponin, complete blood count/complete metabolic panel (CBC/CMP) etc. were reviewed. We characterized cancer patients in terms of ECG patterns, cardiac biomarkers, and echocardiographic findings, including apical vs. midcavitary TTS morphology. Patients with stenosis on quantitative coronary angiography (>50% of left main or >70% of any major coronary artery) or with definite evidence of myocardial infarction were excluded. Additionally, follow-up echocardiography was performed and recovery time to normal left ventricular ejection fraction (LVEF) or, if available, baseline LVEF, was noted.

We compared the overall incidence of major adverse cardiac events, defined as cardiac death or arrest, myocardial infarction, or re-hospitalization for unstable or progressive angina, and overall survival (OS) in cancer patients with TTS, as well as grouped based on triggering event, CI-TTS vs. PE-TTS vs. IM-TTS. The cause of death was classified as cardiac- or cancer-related. Cancer was considered the cause of death if the patient’s demise was secondary to cancer therapy–related complications (e.g., sepsis) or to progression of disease with a patient-requested do not resuscitate/intubate order and/or care transferred to a hospice team.

Additionally, we assessed the impact of the clinical measures of TTS severity, such as LVEF at presentation, cardiac biomarkers or thrombocytopenia, on the prognosis of these groups.

### Statistical analysis

Patient characteristics were summarized with descriptive statistics for the entire group by survival status. Overall survival (OS) was defined as the time interval from the TTS event to death. For survival analysis up to 72 months, patients who were alive were censored at last follow-up or 72 months, whichever was earlier. Patients who died more than 72 months after the TTS event were censored at 72 months. Univariable Cox proportional hazards regression was used to identify factors that were significantly associated with risk of death. Factors identified by this analysis with a *p*-value less than 0.15 were initially included in a multivariable Cox regression model and reduced by backward elimination. Factors with *p*-value less than 0.05 were retained in the multivariable model. A *p*-value of less than 0.05 was regarded as indicating statistical significance. The SAS 9.4 software (SAS Institute INC., Cary, NC) was used for data analysis.

## Results

### Baseline characteristics

Of 373 patients presenting with ACS, 81 (21.7%) patients met the Mayo criteria for diagnosis of TTS and were included in the study. The majority of patients were women (64/81; 79%). Baseline demographics and results of clinical evaluation of TTS patients in the study are shown in Table 1.

**TABLE 1:** Baseline characteristics of the studied population.

		Count (%) or Mean ± SD, Median (range)
Age (years)		65.8 ± 9.3
Creatinine (mg/dl)		0.93 ± 0.35
Platelet count (K/μl)		212.71 ± 151.61
Age (years)	<65	34 (42%)
	≥65	47 (58%)
Sex (*n*)	F	64 (79%)
	M	17 (21%)
Hypertension (*n*)		56 (69.1%)
Dyslipidemia (*n*)		35 (43.2%)
Diabetes mellitus (*n*)		19 (23.4%)
Stroke (*n*)		7 (8.6%)
Coronary artery disease (*n*)		10 (12.3%)
Smoking (*n*)		21 (25.9%)
Heart failure with reduced ejection fraction (*n*)		3 (3.7%)
Malignancy (*n*)	Hematologic	21 (25.9%)
	Solid	60 (74.1%)
Triggering event (*n*)	Chemotherapy	23 (28.5%)
	Medical	22 (27.2%)
	Procedure	18 (22.2%)
	Emotional	7 (8.6%)
	Immunomodulators	7 (8.6%)
	Radiation to Chest	4 (4.9%)
Mechanism (*n*)	Adrenergic	26 (65%)
	Vasospastic	14 (35%)
Morphology (*n*)	Apical	53 (65.4%)
	Midcavitary	27 (33.3%)
Left ventricle ejection fraction on presentation	<30%	15 (18.5%)
	30–39%	24 (29.7%)
	40% and above	42 (51.8%)

Furthermore, for CI-TTS patients, information on primary cancer type, ECG findings, peak troponin I and BNP values, LVEF, angiography findings, TTS morphology (apical/midcavitary), and survival at 72 months are summarized (Supplementary Table 1 and Supplementary Figures 1, 2). Clinical features were further analyzed according to proposed TTS mechanism between (PE-TTS and CI-TTS groups) in Table 2.

**TABLE 2:** Demographic information for the study group organized by proposed TTS mechanism (PEM-TTS vs. CI-TTS).

		PE-TTS (*N* = 26) mean ± SD or count (%)	CI-TTS (*N* = 14) mean ± SD or count (%)
Age (years)		64.23 ± 9.4	66.93 ± 10.25
Creatinine level (mg/dl)		0.8 ± 0.23	0.87 ± 0.45
Platelet count (K/μl) Mean ± SD		219.54 ± 131.21	222.57 ± 196.13
Age (years)	<65	13 (50%)	6 (42.9%)
	≥65	13 (50%)	8 (57.1%)
Sex (*n*)	F	18 (69.2%)	13 (92.9%)
	M	8 (30.8%)	1 (7.1%)
Hypertension (*n*)	No	8 (30.8%)	4 (28.6%)
	Yes	18 (69.2%)	10 (71.4%)
Dyslipidemia (*n*)	No	12 (46.2%)	5 (35.7%)
	Yes	14 (53.8%)	9 (64.3%)
Diabetes mellitus (*n*)	No	21 (80.8%)	11 (78.6%)
	Yes	5 (19.2%)	3 (21.4%)
History of myocardial infarction (*n*)	No	25 (96.2%)	12 (85.7%)
	Yes	1 (3.8%)	2 (14.3%)
Coronary artery disease (*n*)	No	24 (92.3%)	12 (85.7%)
	Yes	2 (7.7%)	2 (14.3%)
Smoking (*n*)	No	14 (53.8%)	10 (71.4%)
	Yes	12 (46.2%)	4 (28.6%)
Malignancy (*n*)	Hematologic	6 (23.1%)	3 (21.4%)
	Solid	20 (76.9%)	11 (78.6%)
TTS Type (*n*)	Apex	14 (53.8%)	12 (85.7%)
	Mid	12 (46.2%)	2 (14.3%)
Echo LVEF	<30%	3 (15.8%)	3 (30%)
	30–39%	7 (36.8%)	3 (30%)
	40–49%	5 (26.3%)	2 (20%)
	≥50%	4 (21.1%)	2 (20%)

CI-TTS, chemotherapy-induced Takotsubo syndrome; PE-TTS, procedural/emotional Takotsubo syndrome; LVEF, left ventricular ejection fraction.

### Results based on triggers

**FIGURE 1:**
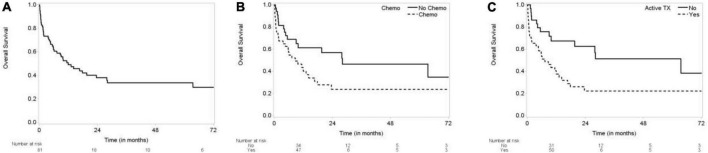
Kaplan-Meier curves for overall survival. **(A)** Overall survival (OS) of TTS patients. **(B)** OS by chemotherapy. **(C)** OS by active treatment.

We observed 23 out of 81 patients (28.5%) had CI-TTS, 22 due to medical condition (27.2%), 18 procedure related (22.2%), 7 (8.6%) due to emotional trigger, 7 (8.6%) due to immunomodulators and 4 (4.9%) due to chest radiation. Majority of the patients were women (79%). The median follow-up time was 31.3 month and median survival was 11.9 months (Figure 1A). All deaths were secondary to cancer and no major adverse cardiac events were recorded during the follow-up period.

Compared to patients not receiving active chemotherapy, the overall survival of patients receiving chemotherapy was worse at 72 months (log-rank test *p* = 0.0405) (Figure 1B). Survival of patients not receiving any kind of cancer therapy (chemo, radiation, etc.) was also significantly better at 72 months (log-rank test *p* = 0.0061) (Figure 1C).

**FIGURE 2:**
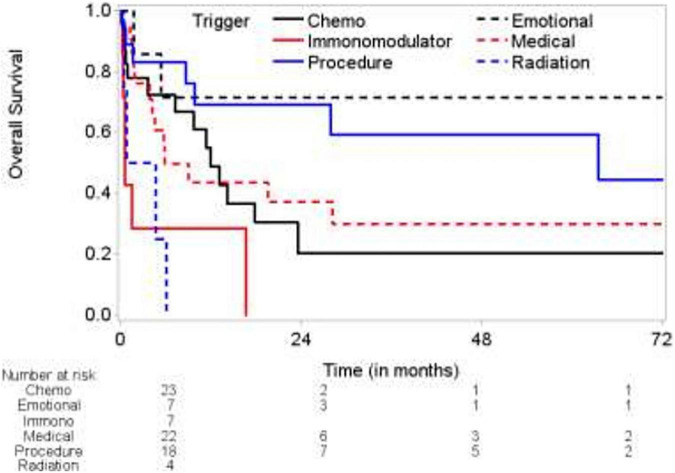
Kaplan-Meier curves for survival based on individual triggers.

Using emotional trigger as reference, Immunomodulator triggered IM-TTS (immune checkpoint inhibitors, notch inhibitors, colony stimulating factors, Anti CD 20, mTOR inhibitor) was associated with HR of 9.7 compared to emotional trigger (CI 1.92–49.1 *p* = 0.0060). Radiation triggered TTS also had HR of 10.369 (1.845–58.264) (*p* = 0.0079). Kaplan-Meier curve comparing the overall survival of patients showed significantly worse prognosis for radiation exposure and immunomodulators with 100% mortality before 24 months (*p* = 0.0004) (Figure 2).

**FIGURE 3:**
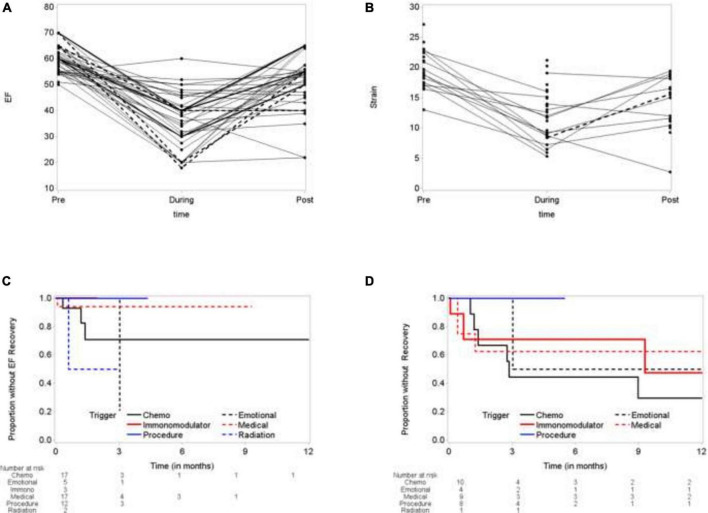
Time to recovery of left ventricular ejection fraction (LVEF) and global longitudinal strain (GLS). **(A)** Election fraction (EF) comparing baseline, during TTS episode and following recovery. **(B)** Global longitudinal strain (GLS) at baseline, during TTS episode and following recovery. **(C)** Time to EF recovery. **(D)** Time to GLS Recovery.

Using paired-*t*-test, the mean ejection fraction prior to TTS event, at the time and during recovery were compared. The mean difference between ejection fraction during TTS and baseline ejection fraction was –21.73 ± 12.93 (*p* < 0.0001), and the mean ejection fraction recovery was 14.83 ± 14.60 (*p* < 0.0001). Despite recovery of ejection fraction, the recovered ejection fraction was –8.93 ± 11.83 (*p* < 0.0001) compared to baseline (Figure 3A and Table 3). Similarly, global longitudinal strain (GLS) was –19.6 ± 3.18% at baseline. During TTS, the mean strain was –11.94 ± 4.21% with a mean difference of 9.04 ± 4.14% (*P* < 0.0001). GLS recovered by a mean difference of –3.63 ± 5.16% following TTS episode (*p* = 0.0420) (Figure 3B and Table 4).

**TABLE 3:** Ejection fraction prior to TTS, during episode and following recovery.

Time	N	Mean ± SD	Comparison	*n*	*P*-value	Diff Mean ± SD
Pre	49	59.41 ± 5.5	During vs. Pre	49	<0.0001	–21.73 ± 12.93
During	81	39.03 ± 11.88	Post vs. During	57	<0.0001	14.83 ± 14.60
Post	57	52.72 ± 10.47	Post vs. Pre	36	<0.0001	–8.93 ± 11.83

**TABLE 4:** Global longitudinal strain during episode and following recovery.

Time	*N*	Mean ± SD	Comparison	*n*	*P*-value	Diff Mean ± SD
Pre	21	–19.6 ± 3.18	During vs. pre	13	<0.0001	9.04 ± 4.14
During	31	–11.94 ± 4.21	Post vs. during	10	0.0420	–3.63 ± 5.16
Post	25	–14.56 ± 4.13	Post vs. pre	7	0.0564	4.83 ± 5.42

Time to EF recovery was dependent on trigger. Chest radiation induced TTS patients showed 50% recovery at 3 month, compared to 80% recovery for emotionally triggers TTS. TTS due to the patient’s medical condition showed the worst recovery (5% at 9 months), followed by chemotherapy induced TTS (25% at 12 months) (Figure 3C). Similarly, time to GLS recovery varied base on trigger. At 12 months, 50% IM-TTS, 70% CI-TTS, 50% emotionally triggered TTS, and 40% of TTS due to patient’s medical condition showed GLS recovery (Figure 3D).

## Discussion

The important findings of this study include, (1) There is a high prevalence (21.7%) of TTS in cancer patients presenting with ACS due to presence of “cancer specific triggers” (use of chemotherapy, immunotherapy and radiation) in addition to traditional triggers (procedure, emotional and medical triggers), (2) In cancer patients with TTS, the survival differed based on inciting trigger with emotional trigger having best survival and immunomodulators and chest radiation triggers having worst survival. (3) Recovery of LVEF also varied based on the event leading to TTS with emotional trigger showing best improvement while medical triggers followed by chemotherapy triggers showing least recovery in LVEF. (4) Recovery of GLS was best with chemotherapy related TTS while medical triggers showing least recovery.

The prevalence of TTS in all patients presenting with ACS has been reported to be 4.4% in one study and around 1–2.5% in a systematic review (22). Prior studies have reported a higher prevalence of malignancy in TTS patients (23). While explicitly studying cancer patients presenting with ACS, a higher prevalence of TTS was observed in our study (21.7%) than previously reported for general patient population (24). Various potential explanations for this observation include emotional turmoil of cancer diagnosis, physical distress from cancer and its treatment and importantly the well-known cardiotoxic effects of various anticancer therapies (24).

In previous large scale studies of TTS patients, physical/medical triggers are more common compared to emotional triggers and independent predictor of in-hospital complications. Our study is unique since we included only cancer patients and concluded that cancer treatment related TTS, particularly immunotherapy or radiation therapy, portrays a worse prognosis when compared to emotional triggers (23). Another important observation was that patients who developed TTS while on treatment with anticancer therapy including chemotherapy, had a worse survival when compared with those who were not receiving active treatment. This may be due to selection bias where more sick patients were exposed to anticancer therapies including chemotherapy that lead to a worse survival.

Two prevailing theories to explain the pathophysiology of TTS include an “excess catecholamine release” and a “transient vasospastic state” (24–27). Among cancer patients with TTS, PE-TTS can be due to various physical stressors like cancer surgery, increased predisposition to infection and sepsis from an immunocompromised state, via an “excess catecholamine release” (24, 25). Vascular endothelial dysfunction in the setting of malignancy and its treatment can explain the CI and IM-TTS via the “transient vasospastic state” (28). Many chemotherapeutics and immunomodulators have been associated with TTS but 5-FU is frequently involved trigger in CI-TTS (29, 30). Recent evidence suggests that inflammation of the coronary adventitial vasa vasorum and perivascular adipose tissue is associated with coronary spasm (31). These results have not yet been replicated in a cancer population; however, cancer is known to be a pro-inflammatory state. Future studies should further evaluate a possible pathophysiological interconnection between cancer—inflammation—vasospasm—TTS. Some other contributing factors including a protective effect of estradiol on the electric disturbances seen in TTS patients and hyperthyroidism seen in TTS patients can be worthwhile targets in treating and preventing TTS in all patients including cancer patients (32, 33).

Another important finding from our study was regarding recovery of LV systolic function. It is now well known from various studies that despite normal or recovered LVEF, patients with TTS can have subclinical LV dysfunction in form of reduced apical or global longitudinal strain (34, 35). In our study CI-TTS patients showed best recovery of GLS while medical triggers had the least recovery of GLS. This could either be due to choosing alternative therapies with minimal cardiotoxic affects in patients developing CI-TTS, as well as meticulous surveillance, and prophylactic use of cardio-protective medications in such patients while on chemotherapy, rendering a better improvement in GLS. Despite a small sample size and being underpowered for this outcome, the improvement in GLS did not correlate with improvement in LVEF during the follow-up.

Having a large population of patients with chemotherapy-induced TTS was useful for noting features of chemotherapy-induced TTS (Supplementary Table 1). We found that patients can present with certain ECG findings. T-wave inversion was the most common ECG pattern in our population, which is consistent with our previous study (23, 24). Among patients with exposure to chemotherapy, the apical morphology was found in the vast majority of cases, while the distribution between apical and midcavitary morphology was more equal in the PE-TTS group. Unfortunately, our study was not powered to detect statistical significance of these features in CI-TTS. Moreover, limited data exists regarding rechallenging patients with cancer therapy induced TTS with the same therapy. Rechallenging should be avoided if possible, particularly if the LVEF or GLS does not completely normalize (21). If it is inevitable or if the LVEF and GLS have normalized, rechallenging can be considered while maintaining patients on the cardioprotective treatments and with close surveillance (36).

### Strengths and limitations

Several aspects of our study contribute to its strength. First, we had access to a large population of cancer patients with TTS and the largest population of patients with CI-TTS known to date. Second, patients included in our study who are not classified as having CI-TTS are, nonetheless, cancer patients, eliminating a potential confounding factor if CI-TTS were to be compared with the TTS population in general. Third, the cardiac catheterization laboratory at The University of Texas MD Anderson Cancer Center allowed for on-site documentation of absence of prior cardiac disease and TTS morphology with angiography during the acute TTS episode. This advantage allowed our unique patient population to undergo state-of-the-art cancer treatment as well as cardiac assessment within the same facility. An important aspect for future studies can involve studying involvement of right ventricle (RV) function including RV strain to assess for subset of patients who present with biventricular TTS. Data regarding RV function during the episode of TTS at follow-up was not obtained in this study but prior studies have indicated a higher prevalence of biventricular TTS in cancer patients (37).

On the other hand, our study suffers from the known limitations of retrospective study. Additionally, a causal relationship between various TTS triggers and survival is extremely difficult to establish due to multiple confounding factors in this complex patient population. Despite the higher incidence of TTS in cancer patients, the sample size of patients is simply not large enough to produce a study with matching that may point to chemotherapy as the only factor causing worse outcomes in these patients. Nonetheless, we believe it is important for clinicians to note that patients with TTS who have recent exposure to chemotherapy are at higher risk for death extending beyond the duration of cancer treatment. Identification of the triggering event in cancer patients with TTS also presents the challenge of separating pure emotional stress from the psychological and physical stress that parallels diagnosis, treatment, and follow-up of malignancies. Also, distinguishing LV dysfunction from myocardial toxicity from CI-TTS can be challenging in the absence of endomyocardial biopsy and is strictly based on the left ventriculogram and echocardiographic appearance (cardiotoxicity with a global and more patchy appearance, vs. CI–TTS with the typical subtypes: apical, midcavitary, or reversed). Our classification of the mechanism of TTS in this study is empirical, since it is possible that both catecholamine excess and vasospasm are involved in the pathogenesis of both CI-TTS and PE-TTS. Unfortunately, the number of patients who qualify for a study on CI-TTS will always be low, and thus the study suffers from limitations in terms of the power of its findings. It is important to note that the distribution of cancers involved in this study could differ significantly from that of another population of patients. Different malignancies can have significantly varied prevalence, treatments, and outcomes.

## Conclusion

TTS can present in cancer patients after a wide spectrum of triggering events. CI-TTS presents mainly in women and is predominantly segmental and apical in morphology on echocardiography. PE-TTS appears to be better tolerated and to have better outcomes at 2 years. Underlying mechanisms of TTS could possibly predict outcomes in a cancer population. These results suggest that identifying a physical or emotional stressor as a cause of TTS among cancer patients may indicate a better prognosis than TTS induced by immunotherapy, radiation therapy and chemotherapy.

## Data availability statement

The raw data supporting the conclusions of this article will be made available by the authors, without undue reservation.

## Ethics statement

The studies involving human participants were reviewed and approved by the MD Anderson Institutional Review Board. Written informed consent for participation was not required for this study in accordance with the national legislation and the institutional requirements.

## Author contributions

AS and TA contributed to conception or design of the work and the acquisition, analysis or interpretation of data for the work, and drafting the work or revising it critically for important intellectual content. VL and AAd contributed to the acquisition, analysis or interpretation of data for the work, and drafting the work. AAg contributed to revising it critically for important intellectual content. DG and DB did analysis of the data and provided approval for publication of the content. TDo, TDa, JL-M, PK, SH, KK, NP, JT, GI, EY, JH, KM, and PA contributed to revising it critically for important intellectual content and final approval. CI contributed to revising it critically, provided final approval of publication and agreed to be accountable for all aspects of the work in ensuring that questions related to the accuracy or integrity of any part of the work are appropriately investigated and resolved. All authors contributed to the article and approved the submitted version.

## Supplementary material

Supplementary Figure 1 Diagnostic studies for chemotherapy induced apical takotsubo syndrome.

Supplementary Figure 2 Diagnostic studies for procedural/emotional trigger induced midcavitary takotsubo syndrome.

Supplementary Table 1 Showing characteristics of various chemotherapy regimen induced takotsubo syndrome.
