# Supplementary figures and images for: Trigger related outcomes of takotsubo syndrome in a cancer population
Source: Front Cardiovasc Med. 2022 Oct 28;9:1019284. doi: 10.3389/fcvm.2022.1019284 (PMC9651211; doi:10.3389/fcvm.2022.1019284)

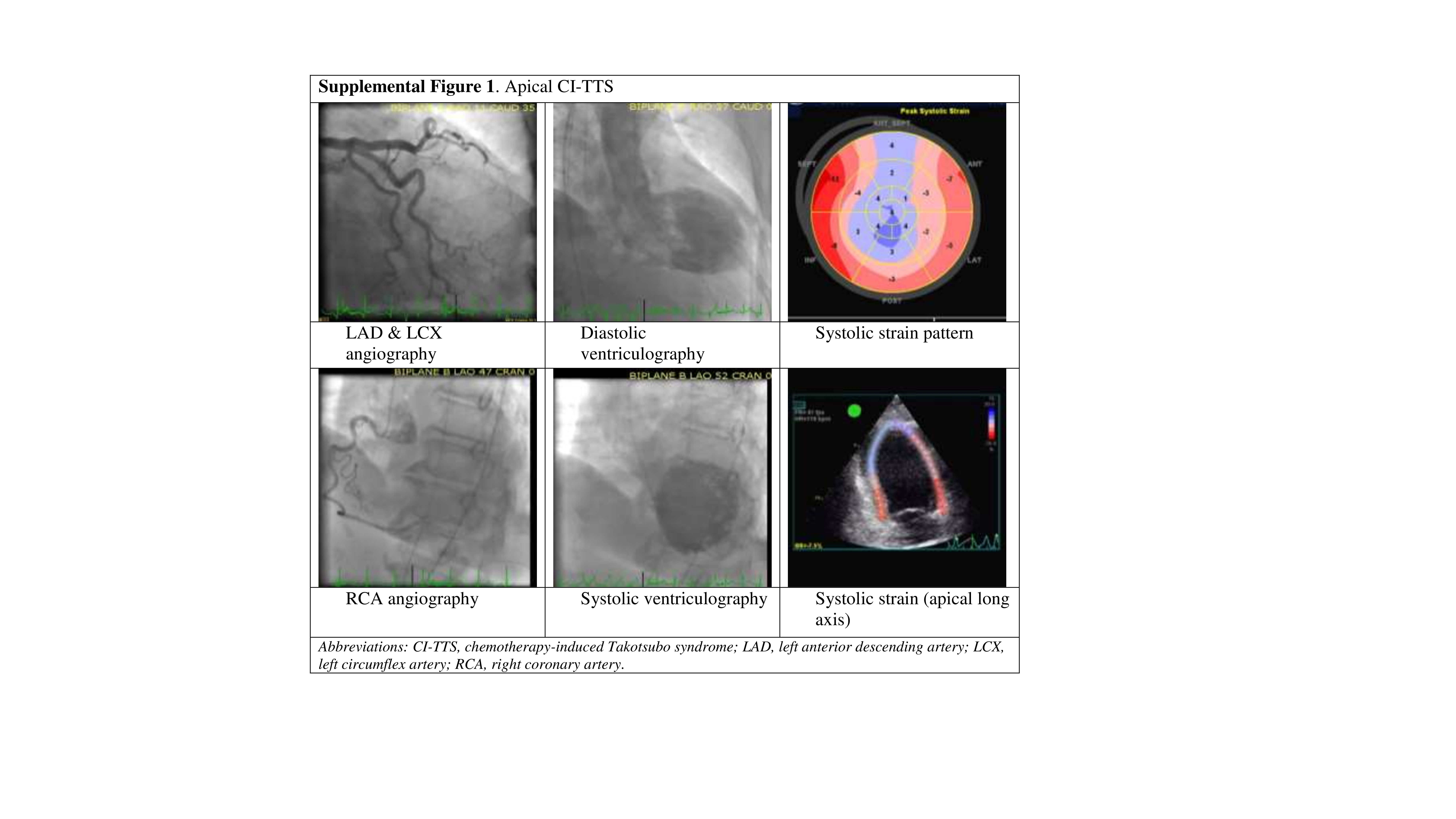

Supplement: Supplementary Figure 1 — Diagnostic studies for chemotherapy induced apical takotsubo syndrome. [file Image_1.tiff]

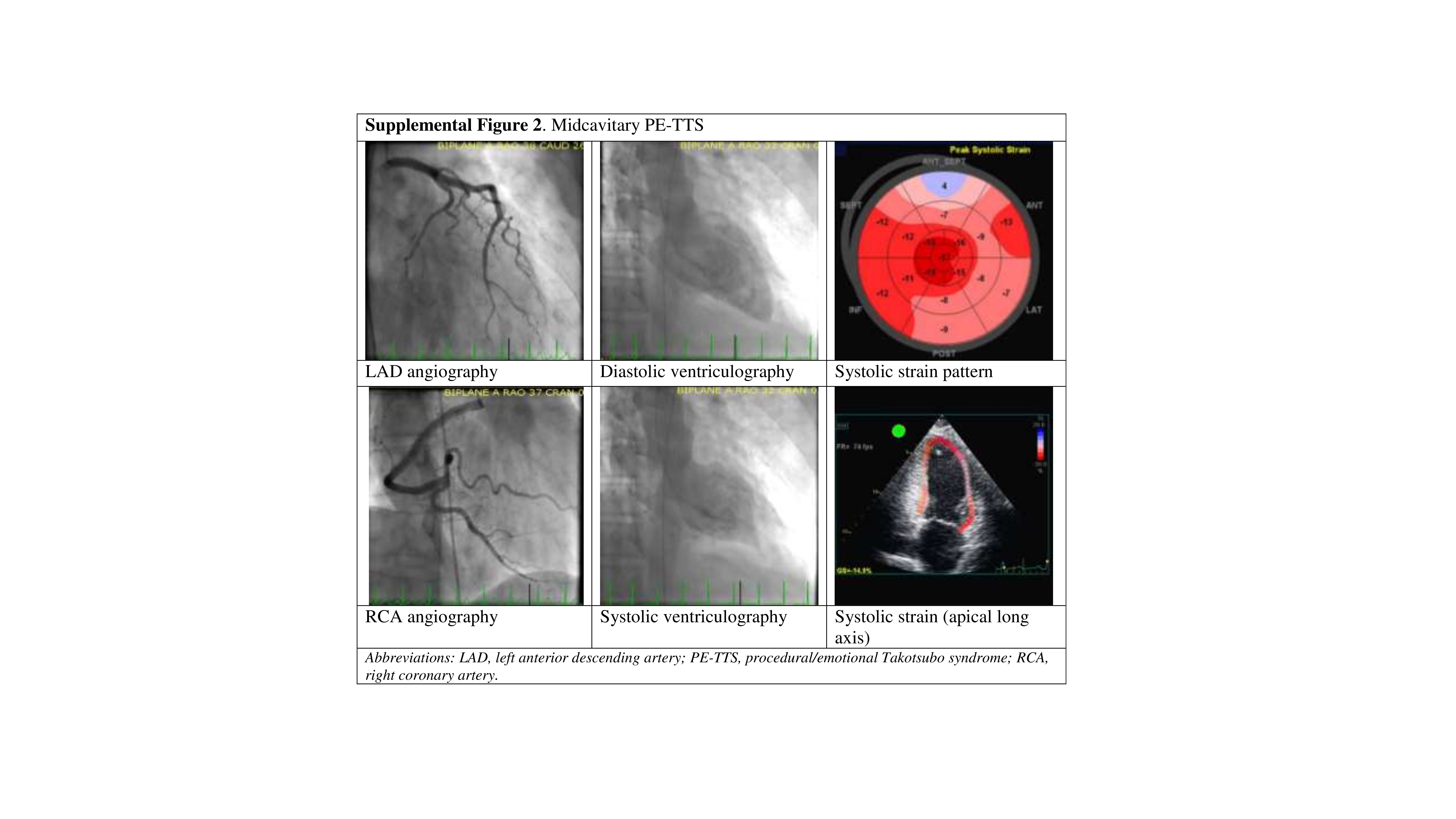

Supplement: Supplementary Figure 2 — Diagnostic studies for procedural/emotional trigger induced midcavitary takotsubo syndrome. [file Image_2.tiff]
