# Supplementary material for: Trigger related outcomes of takotsubo syndrome in a cancer population
Source: Front Cardiovasc Med. 2022 Oct 28;9:1019284. doi: 10.3389/fcvm.2022.1019284 (PMC9651211; doi:10.3389/fcvm.2022.1019284)
Supplement: Supplementary Table 1 — Showing characteristics of various chemotherapy regimen induced takotsubo syndrome. [file Table_1.docx]

| **No.** | **Age (years)** | **Sex** | **Cancer type** | **Chemotherapy regimen** | **ECG finding** | **Peak TnI (ng/mL)** | **Peak BNP (pg/mL)** | **Echo LVEF** | **Angiography** | **Type** | **Survival (months)** | | |
| --- | --- | --- | --- | --- | --- | --- | --- | --- | --- | --- | --- | --- | --- |
|  |  |  |  |  |  |  |  |  |  |  | **6** | **12** | **24** |
| 1. | 70 | M | Metastatic prostate cancer | Paclitaxel | T wave inversion | 1.8 | 555 | 32.5% | 30% LAD | Apical | Yes | No | No |
| 2. | 72 | F | Ovarian carcinoma | Paclitaxel | T wave inversion | 1.47 | 1525 | 43.8% | 60% RCA | Apical | Yes | No | No |
| 3. | 77 | F | Ovarian carcinoma | Carboplatin, Paclitaxel | T wave inversion | 0.82 | 2299 | 28.9% | Luminal irregularities | Apical | Yes | Yes | No |
| 4. | 77 | F | Ovarian carcinoma | Carboplatin, Paclitaxel | T wave inversion | 0.75 | 50 | 31.2% | Luminal irregularities | Apical | No | No | No |
| 5. | 65 | F | Gastric adenocarcinoma | Oxaliplatin, 5-FU | T wave inversion |  |  | 35.0% | Luminal irregularities | Apical | Yes | Yes | No |
| 6. | 61 | F | Appendiceal carcinoma | Folinic acid, 5-FU, Oxaliplatin | Sinus tachycardia | 1.06 | 1502 | 20.4% | Luminal irregularities | Apical | Yes | Yes | No |
| 7. | 81 | F | Pancreatic adenocarcinoma | 5-FU, Gemcitabine | ST elevation | 0.7 | 300 | unchanged | Normal | Apical | Yes | Yes | Has not reached |
| 8. | 58 | F | Gallbladder adenocarcinoma | Bevacizumab, Irinotecan, Capecitabine | ST elevation | 4.45 | 2643 | 20.2% | Luminal irregularities | Apical | No | No | No |
| 9. | 73 | F | AML | Decitabine, ABT-199* | ST elevation | 4.34 | >4700 | 31.0% | Luminal irregularities | Apical | Yes | No | No |
| 10. | 58 | F | Thymoma | Cyclophosphamide, Doxorubicin, Cisplatin | Normal sinus rhythm | 1.21 | 651 | 46.0% | Normal | Mid | Yes | Yes | Has not reached |
| 11. | 51 | F | Leiomyosarcoma | Doxorubicin, Dacarbazine, Pegfilgrastin | ST elevation | 3.3 | 56 | 34.0% | Normal | Apical | No | No | No |
| 12. | 53 | F | EGFR-resistant NSCLC | Ibrutinib | ST elevation | 0.68 | 77 | 35.0% | Normal | Mid | No | No | No |
| 13. | 81 | F | MDS | Nivolumab, Ipilimumab | T wave inversion | 0.08 | 728 | 50.0% | Normal | Apical | No | No | No |
| 14. | 60 | F | AML | MDM2 inhibitor | T wave inversion | 0.03 | 3049 | 32.0% | Luminal irregularities | Apical | Yes | No | No |
| **BCL-2 inhibitor*  *Abbreviations: 5-FU, 5-fluorouracil; AML, acute myeloid leukemia; BCL-2, B cell lymphoma 2; BNP, brain natriuretic peptide; ECG, electrocardiogram; EGFR, epidermal growth factor receptor; LAD, left anterior descending artery; LVEF, left ventricular ejection fraction; MDM2, murine double minute 2; MDS, myelodysplastic syndrome; NSCLC, non–small cell lung cancer; RCA, right coronary artery; SOB, shortness of breath; TnI, troponin I.* | | | | | | | | | | | | | |
